# Supplementary material for: Increasing Fruit and Vegetable Variety over Time Is Associated with Lower 15-Year Healthcare Costs: Results from the Australian Longitudinal Study on Women’s Health
Source: Nutrients. 2021 Aug 18;13(8):2829. doi: 10.3390/nu13082829 (PMC8398554; doi:10.3390/nu13082829)
Supplement: Supplementary file 1 [file nutrients-13-02829-s001.zip › nutrients-1300018-supplementary.pdf]

Supplemental Table S1. Modified FAVVA index.

<u>The Modified FAVVA index: Total score = 185</u>	Scoring	Maximum score
**1 Vegetables**		
**F1. How many different vegetables do you usually eat per day?**		
O <1 per day<br>O 1 per day<br>O 2 per day<br>O 3 per day<br>O 4 per day<br>O 5 per day<br>O 6+ per day	0<br>1<br>2<br>3<br>4<br>5<br>6	6
**F2. Potato boiled, mashed, baked (1 serving)**		
O Never<br>O Less than 1 per month<br>O 1-3 per month<br>O Once per week<br>O 2-4 per week<br>O 5 or more per week	0<br>1<br>2<br>3<br>4<br>5	5
**F3. Pumpkin (1 serving)**		
O Never<br>O Less than 1 per month<br>O 1-3 per month<br>O Once per week<br>O 2-4 per week<br>O 5 or more per week	0<br>1<br>2<br>3<br>4<br>5	5
**F4. Cucumber**		
O Never<br>O Less than 1 per month<br>O 1-3 per month<br>O Once per week<br>O 2-4 per week<br>O 5 or more per week	0<br>1<br>2<br>3<br>4<br>5	5
**F5. Cauliflower (1 serving)**		
O Never<br>O Less than 1 per month<br>O 1-3 per month<br>O Once per week<br>O 2-4 per week<br>O 5 or more per week	0<br>1<br>2<br>3<br>4<br>5	5
**F6. Green beans (1 serving)**		
O Never<br>O Less than 1 per month<br>O 1-3 per month<br>O Once per week<br>O 2-4 per week<br>O 5 or more per week	0<br>1<br>2<br>3<br>4<br>5	5
**F7. Spinach (1 serving)**		
O Never<br>O Less than 1 per month<br>O 1-3 per month<br>O Once per week<br>O 2-4 per week<br>O 5 or more per week	0<br>1<br>2<br>3<br>4<br>5	5
**F8. Cabbage or brussel sprouts (1 serving)**		

O Never<br>O Less than 1 per month<br>O 1-3 per month<br>O Once per week<br>O 2-4 per week<br>O 5 or more per week	0<br>1<br>2<br>3<br>4<br>5	5
**F9. Peas (1 serving)**		
O Never<br>O Less than 1 per month<br>O 1-3 times per month<br>O Once per week<br>O 2-4 times per week<br>O 5-6 times per week<br>O Once per day<br>O 2 or more times per day	0<br>1<br>2<br>3<br>4<br>5<br>6<br>7	7
**F10. Broccoli (1 serving)**		
O Never<br>O Less than 1 per month<br>O 1-3 times per month<br>O Once per week<br>O 2-4 times per week<br>O 5-6 times per week<br>O Once per day<br>O 2 or more times per day	0<br>1<br>2<br>3<br>4<br>5<br>6<br>7	7
**F11. Carrots (1 serving)**		
O Never<br>O Less than 1 per month<br>O 1-3 times per month<br>O Once per week<br>O 2-4 times per week<br>O 5-6 times per week<br>O Once per day<br>O 2 or more times per day	0<br>1<br>2<br>3<br>4<br>5<br>6<br>7	7
**F12. Zucchini, eggplant, squash (1 serving)**		
O Never<br>O Less than 1 per month<br>O 1-3 per month<br>O Once per week<br>O 2-4 per week<br>O 5 or more per week	0<br>1<br>2<br>3<br>4<br>5	5
**F13. Capsicum (1 serving)**		
O Never<br>O Less than 1 per month<br>O 1-3 per month<br>O Once per week<br>O 2-4 per week<br>O 5 or more per week	0<br>1<br>2<br>3<br>4<br>5	5
**F14. Mushroom (1 serving)**		
O Never<br>O Less than 1 per month<br>O 1-3 per month<br>O Once per week<br>O 2-4 per week<br>O 5 or more per week	0<br>1<br>2<br>3<br>4<br>5	5
**F15. Tomatoes (1 serving)**		
O Never<br>O Less than 1 per month	0<br>1<br>2	5

O 1-3 per month<br>O Once per week<br>O 2-4 per week<br>O 5 or more per week	3<br>4<br>5	
**F16. Lettuce (1 serving)**		
O Never<br>O Less than 1 per month<br>O 1-3 times per month<br>O Once per week<br>O 2-4 times per week<br>O 5-6 times per week<br>O Once per day<br>O 2 or more times per day	0<br>1<br>2<br>3<br>4<br>5<br>6<br>7	7
**F17. Celery (1 serving)**		
O Never<br>O Less than 1 per month<br>O 1-3 per month<br>O Once per week<br>O 2-4 per week<br>O 5 or more per week	0<br>1<br>2<br>3<br>4<br>5	5
**F18. Avocado (1 serving)**		
O Never<br>O Less than 1 per month<br>O 1-3 per month<br>O Once per week<br>O 2-4 per week<br>O 5 or more per week	0<br>1<br>2<br>3<br>4<br>5	5
**F19. Onion, spring onion, leek (1 serving)**		
O Never<br>O Less than 1 per month<br>O 1-3 per month<br>O Once per week<br>O 2-4 per week<br>O 5 or more per week	0<br>1<br>2<br>3<br>4<br>5	5
**F20. Soybeans, tofu (1 serving)**		
O Never<br>O Less than 1 per month<br>O 1-3 per month<br>O Once per week<br>O 2-4 per week<br>O 5 or more per week	0<br>1<br>2<br>3<br>4<br>5	5
**F21. Baked beans (1 serving)**		
O Never<br>O Less than 1 per month<br>O 1-3 per month<br>O Once per week<br>O 2-4 per week<br>O 5 or more per week	0<br>1<br>2<br>3<br>4<br>5	5
**F22. Other beans, lentils e.g. chick peas, split peas (1 serving)**		
O Never<br>O Less than 1 per month<br>O 1-3 per month<br>O Once per week<br>O 2-4 per week<br>O 5 or more per week	0<br>1<br>2<br>3<br>4<br>5	5
**Fruit**		
**F23. How many pieces of fruit do you eat per day (include all types)**		

O None<br>O <1 piece per day<br>O 1 piece/day<br>O 2 pieces/day<br>O 3 pieces per day<br>O 4+ pieces per day	0<br>1<br>2<br>3<br>4<br>5	5
**F24. Canned/frozen fruit eg. peaches, two fruits (1 serving)**		
O Never<br>O Less than 1 per month<br>O 1-3 per month<br>O Once per week<br>O 2-4 per week<br>O 5 or more per week	0<br>1<br>2<br>3<br>4<br>5	5
**F25. Pear (1 serving)**		
O Never<br>O Less than 1 per month<br>O 1-3 per month<br>O Once per week<br>O 2-4 per week<br>O 5 or more per week	0<br>1<br>2<br>3<br>4<br>5	5
**F26. Apricot (1 serving)**		
O Never<br>O Less than 1 per month<br>O 1-3 per month<br>O Once per week<br>O 2-4 per week<br>O 5 or more per week	0<br>1<br>2<br>3<br>4<br>5	5
**F27. Apple (1 serving)**		
O Never<br>O Less than 1 per month<br>O 1-3 times per month<br>O Once per week<br>O 2-4 times per week<br>O 5-6 times per week<br>O Once per day<br>O 2 or more times per day	0<br>1<br>2<br>3<br>4<br>5<br>6<br>7	7
**F28. Orange, mandarin, grapefruit (1 serving)**		
O Never<br>O Less than 1 per month<br>O 1-3 times per month<br>O Once per week<br>O 2-4 times per week<br>O 5-6 times per week<br>O Once per day<br>O 2 or more times per day	0<br>1<br>2<br>3<br>4<br>5<br>6<br>7	7
**F29. Banana (1 serving)**		
O Never<br>O Less than 1 per month<br>O 1-3 times per month<br>O Once per week<br>O 2-4 times per week<br>O 5-6 times per week<br>O Once per day<br>O 2 or more times per day	0<br>1<br>2<br>3<br>4<br>5<br>6<br>7	7
**F30. Peach, nectarine, plum or apricot (1 serving)**		
O Never<br>O Less than 1 per month<br>O 1-3 per month<br>O Once per week	0<br>1<br>2<br>3	5

O 2-4 per week<br>O 5 or more per week	4<br>5	
**F31. Mango or paw-paw (1 serving)**		
O Never<br>O Less than 1 per month<br>O 1-3 per month<br>O Once per week<br>O 2-4 per week<br>O 5 or more per week	0<br>1<br>2<br>3<br>4<br>5	5
**F32. Pineapple (1 serving)**		
O Never<br>O Less than 1 per month<br>O 1-3 per month<br>O Once per week<br>O 2-4 per week<br>O 5 or more per week	0<br>1<br>2<br>3<br>4<br>5	5
**F33. Strawberries (1 serving)**		
O Never<br>O Less than 1 per month<br>O 1-3 per month<br>O Once per week<br>O 2-4 per week<br>O 5 or more per week	0<br>1<br>2<br>3<br>4<br>5	5
**F34. Melon eg. watermelon, rockmelon (1 serving)**		
O Never<br>O Less than 1 per month<br>O 1-3 per month<br>O Once per week<br>O 2-4 per week<br>O 5 or more per week	0<br>1<br>2<br>3<br>4<br>5	5
	**Maximum score**	**185**

Supplemental Table S2. Differences in methods used to calculate the FAVVA index derived from the AES FFQ and the DQESv2 FFQ.

	**Ashton et al 2018**	**Current study**
**FAVVA Item**	**AES item**	**DQESv2 item**
Overall vegetable intake	Times per week vegetables consumed with evening meal	Number of different vegetables consumed per day
Sweet potato	Never – 5+ per week	*Not assessed*
Corn	Never – 5+ per week	*Not assessed*
Celery, cucumber	Assessed together	Assessed separately (Celery; Cucumber)
Overall fruit intake	8-point scale from None to 4+ per day	6-point scale from None to 4+ per day
Fruit salad	Never – 5+ per week	*Not assessed*
Dried fruit	Never – 5+ per week	*Not assessed*
Apple, pear	Assessed together	Assessed separately (Apples; Pears)
Peach, nectarine, plum, apricot	Assessed together	Assessed separately (Apricots; Peaches/nectarines)
Grapes, strawberries, blueberries	Assessed together	Assessed as Strawberries only
**Maximum score**	190	185

Abbreviations: AES, Australian Eating Survey; DQESv2, Dietary Questionnaire for Epidemiological Studies version 2; FAVVA, Fruit and Vegetable Variety index; FFQ, Food Frequency Questionnaire

Supplemental Table S3. Coefficients and 95% confidence intervals per 100-gram baseline fruit and vegetable intake (g/day) as a predictor of 15-year (2001-2015) cumulative Medicare claims and costs ($AUD) for Australian women born 1946-51, by baseline BMI category (*n*=8833).

**2001 Fruit and vegetable intake**	**Medicare variable**	**Healthy weight** *n*=3743	**Overweight/obese** *n*=4971	**All** *n*=8833
**Fruit and Vegetable intake**	Claims (*n*)[a]	-1.9 (-4.9, 1.0)	**5.1 (2.1, 8.2)***	**2.9 (0.7, 5.0)***
	Charge ($)[b]	-170.0 (-390.2, 50.2)	**323.5 (106.4, 540.6)***	**159.3 (3.8, 314.7)***
	Benefit ($)[c]	-124.4 (-285.0, 36.3)	**269.1 (109.8, 428.4)***	**139.0 (24.8, 253.2)***
	Gap ($)[d]	-45.6 (-122.3, 31.0)	54.4 (-21.4, 130.2)	20.3 (-33.6, 74.1)
**Fruit intake**	Claims (*n*)	-3.0 (-6.9, 0.8)	**7.2 (3.4, 11.1)***	**3.7 (0.9, 6.4)***
	Charge ($)	-167.7 (-455.4, 120.0)	**477.5 (200.1, 754.9)***	**253.6 (52.9, 454.4)***
	Benefit ($)	-155.0 (-364.9, 54.9)	**387.5 (184.0, 591.1)***	**197.2 (49.7, 344.7)***
	Gap ($)	-12.7 (-112.9, 87.4)	89.9 (-6.9, 186.8)	56.4 (-13.1, 126.0)
**Vegetable intake**	Claims (*n*)	-0.8 (-7.5, 5.9)	3.6 (-3.2, 10.5)	3.3 (-1.5, 8.2)
	Charge ($)	-368.3 (-867.4, 130.7)	157.5 (-331.3, 646.4)	36.7 (-314.2, 387.5)
	Benefit ($)	-172.2 (-536.4, 191.9)	161.0 (-197.8, 519.8)	105.8 (-152.0, 363.6)
	Gap ($)	**-196.1 (-369.7, -22.5)***	-3.5 (-174.1, 167.1)	-69.1 (-190.6, 52.4)

*$p<0.05$; linear regression modelling with adjustment for area of residence, ability to manage on current income and total energy intake.
Abbreviations: BMI, body mass index; FAVVA, Fruit and Vegetable Variety index
Note: Women with 'Underweight' BMI were excluded from analysis due to small sample size (*n*=113).
[a] Number of healthcare services received under the Medicare Benefits Schedule
[b] Total cost of services (as charged by healthcare provider)
[c] Amount paid back to the patient by Medicare
[d] Out-of-pocket costs paid by the patient

Supplemental Table S4. Coefficients and 95% confidence intervals (CI) per 100-gram change in daily fruit and vegetable intake (2001-2013) as a predictor of 15-year (2001-2015) cumulative Medicare claims and costs ($AUD) for Australian women born 1946-51, by BMI category (*n*=6955).

**Change in intake 2001-2013**	**Medicare variable**	**Healthy weight** *n*=3007	**Overweight/obese** *n*=3857	**All** *n*=6955
**Fruit and Vegetable intake**	Claims (*n*)[a]	-3.0 (-7.1, 1.1)	**-7.7 (-11.7, -3.7)***	**-6.5 (-9.4, -3.6)***
	Charge ($)[b]	-114.0 (-424.8, 196.8)	**-650.4 (-946.7, -354.1)***	**-480.0 (-694.7, -265.2)***
	Benefit ($)[c]	-157.7 (-383.1, 67.7)	**-458.1 (-673.5, -242.8)***	**-374.2 (-530.7, -217.7)***
	Gap ($)[d]	43.7 (-65.7, 153.0)	**-192.3 (-297.0, -87.5)***	**-105.8 (-181.1, -30.5)***
**Fruit intake**	Claims	-1.1 (-6.2, 4.0)	**-5.2 (-10.3, -0.07)***	**-4.4 (-8.1, -0.7)***
	Charge	-15.6 (-403.6, 372.3)	**-547.5 (-926.4, -168.5)***	**-393.3 (-665.4, -121.3)***
	Benefit	-64.5 (-345.9, 216.9)	**-358.4 (-633.8, -82.9)***	**-288.4 (-486.7, -90.1)***
	Gap	7.0 (-73.0, 207.0)	**-201.7 (-341.2, -62.0)***	**-105.0 (-200.3, -9.6)***
**Vegetable intake**	Claims	**-10.6 (-19.4, -1.9)***	**-20.1 (-28.8, -11.5)***	**-16.7 (-23.0, -10.5)***
	Charge	-477.9 (-1142.0, 186.2)	**-1404.1 (-2047.1, -761.1)***	**-1046.1 (-1508.5, -583.7)***
	Benefit	**-540.3 (-1021.9, -58.7)***	**-1062.1 (-1529.5, -594.7)***	**-872.0 (-1208.9, -535.0)***
	Gap	62.4 (-171.3, 296.0)	**-342.0 (-569.2, -114.8)***	**-174.1 (-336.2, -12.0)***

*$p < 0.05$; linear regression modelling with adjustment for area of residence, ability to manage on current income, total energy intake and baseline fruit and vegetable consumption.
Abbreviations: BMI, body mass index; FAVVA, Fruit and Vegetable Variety index.
Note: Women with 'Underweight' BMI were excluded from analysis due to small sample size (n=91).
[a] Number of healthcare services received under the Medicare Benefits Schedule
[b] Total cost of services (as charged by healthcare provider)
[c] Amount paid back to the patient by Medicare
[d] Out-of-pocket costs paid by the patient
